# Obesity and long term functional outcomes following elective total hip replacement

**DOI:** 10.1186/1749-799X-7-16

**Published:** 2012-04-25

**Authors:** Heather K Vincent, MaryBeth Horodyski, Peter Gearen, Richard Vlasak, Amanda N Seay, Bryan P Conrad, Kevin R Vincent

**Affiliations:** 1 Interdisciplinary Center for Musculoskeletal Training and Research, Department of Orthopaedics and Rehabilitation, Divisions of Research, Joint Reconstruction, & Physical Medicine and Rehabilitation, University of Florida, Gainesville, FL, 32611, USA

**Keywords:** Arthroplasty, Body mass index, Hip, Physical function, Disability, Obesity

## Abstract

**Introduction:**

Obesity rates continue to rise and more total hip arthroplasty procedures are being performed in progressively younger, obese patients. Hence, maintenance of long term physical function will become very important for quality of life, functional independence and hip prosthesis survival. Presently, there are no reviews of the long term efficacy of total hip arthroplasty on physical function. This review: 1) synopsized available data regarding obesity effects on long term functional outcomes after total hip arthroplasty, and 2) suggested future directions for research.

**Methods:**

A literature search was conducted from 1965 to January of 2011 for studies that evaluated long term functional outcomes at one year or longer after THA in obese (body mass index values ≥30 kg/m^2^) and non-obese patients (body mass index <30 kg/m^2^).

**Results:**

Five retrospective studies and 18 prospective studies were identified as those that assessed physical function before surgery out to ≥ one year after total hip arthroplasty. Study sample sizes ranged from 108–18,968 and followed patients from one to twenty years. Total hip arthroplasty confers significant pain reduction and improvement in quality of life irrespective of body mass index. Functional improvement occurred after total hip arthroplasty among all studies, but obese patients generally did not attain the same level of physical function by the follow-up time point.

**Discussion:**

Uncontrolled obesity after total hip arthroplasty is related to worsening of comorbidities and excessive health care costs over the long term. Aggressive and sustainable rehabilitation strategies that include physical exercise, psychosocial components and behavior modification may be highly useful in maximizing and maintaining weight loss after total hip arthroplasty.

## Introduction

The obese segment of the population with osteoarthritis is burgeoning, and the demand for total hip arthroplasty (THA) surgeries to treat obese persons is rising rapidly. Primary THA procedures will likely become necessary for a greater prevalence of the U.S. population who is developing debilitating OA earlier in life, and revision THAs will become more frequent as these young adults age and the life of the joint component ends. Evidence indicates that the relative risk ratio for undergoing elective hip replacement ranges from 1.92 in overweight individuals to 8.56 in severely obese individuals [1]. While extensive evidence has focused on surgical outcomes, mortality medical complications after hip replacement [2], the relationship between functional outcomes and obesity after THA over the long term is not well understood, particularly in the obese patient. This is a significant scientific deficit as restoration of physical function is one of the primary goals of THA [3,4].

As obesity rates continue rising and more THAs are being performed in progressively younger, obese patients, long term functional goals will become important. Functional goals may include maintaining independence with load bearing activities of daily living, independent mobility and body transfers over the long term. Many obese patients with THA will be recommended to perform exercise (heavy demand or weight bearing activity) for weight loss. In addition, progressively more obese patients with THA will be living longer with a joint implant and may be at risk for failure and poorer quality of life over the long duration compared with non-obese patients. For example, 35% of patients with THA experience severe activity limitation by year five; obesity significantly predicted complete dependence on walking aids and is associated with depression at follow-up [5]. The physical component of quality of life is also lower in obese patients years after the procedure [6-8]. Hence, a clear understanding the effect of obesity on functional outcomes after THA will be critical in establishing expectations for the patients and care team and development of strategies to optimize physical function and independent mobility for as long as possible. Therefore, the purposes of this review are to: 1) provide a current synopsis of the available data regarding obesity effects on long term functional outcomes after THA, and 2) to suggest future directions for research.

## Search strategy

We conducted a literature search from 1965 to January of 2011 in Medline, Cochrane Controlled Trials Register, CINAHL, Scopus and Web of Science. The search strategy identified studies in English that examined the relationships between obesity or BMI, physical function after total hip replacement. A functional measurement of mobility, ambulation or transfers or a comprehensive self-report tool that assesses physical function had to have been reported a minimum of six months of follow-up after surgery. Estimates of obesity had to have been reported including body mass index (BMI), body fat percentage or fat mass. Medical subject headings (MeSH)/keywords with all subheadings and as free text included obesity, obese, mobility, functional limitation, physical function, body fat, adiposity, waist circumference, total hip replacement and hip arthroplasty. Studies that administered surveys that reflected the perceived physical functional ability were also included; these tools included the Oxford Hip score (OHS), Harris Hip score (HHS), Western Ontario McMaster Osteoarthritis Index (WOMAC) score or other specially constructed surveys that included function-based questions, such as the University of California Los Angeles (UCLA) hip scoring system. Studies that used functional tests (walking tests, chair rise or timed up and go or body transfers or other self care tasks) were also included. A total of 552 papers were initially generated from this search. Studies that had a follow-up of less than six months, did not report any form of functional measure at follow-up or did not include an assessment of obesity were not included. The lists of references of retrieved publications were manually checked to add any citations missed by the electronic searches. Obesity was defined as an excessive body weight >100 kg, a BMI ≥30 kg/m^2 ^[9], or a large waist circumference (>88 cm women, >102 cm men) [10].

### Characteristics of included studies

A total of 23 articles were reviewed. Twenty articles included self-report assessments of the WOMAC, HHS or OHS or other survey based tools of physical function. Eight studies included objective physical function assessments. Studies were conducted in the United States [7,11-13], Europe (Netherlands [14], Scotland [15], the United Kingdom [6,16-19], Switzerland [3], Denmark [20]), Canada [2,21] and Australia [22-25]. Study sample sizes ranged from 140 [15] to 18,968 [3] and were comprised of 17-77% women, depending on the study. We identified three multicenter studies (two from Britain, one from Switzerland, and one with 7 to 20 sites) [3,16,18] and two studies based on joint surgery registries (American-based registry study) [13] and a Canadian based registry [26].

Several studies demonstrated variability in the follow-up time points for assessment, which limits generalizability of the findings. For example, two retrospective studies captured follow up data from 0.1-15.6 years [14] and from 10–18.9 years [11]. Other retrospective studies captured follow-up times out to one year [2] or at different points of time during the years following the surgery [7,11,24]. A few prospective studies contained follow-up data from different time points during years following the procedure [23,27].

### Hip surgical components and approaches

Surgical technique, component type and use of cement varied among and within the identified studies. Several surgical techniques were used across the studies, including anterolateral [6,14,16,19], posterior [7,16,23], lateral [28] and posterolateral [11,20] approaches. There was also variation in the type of component used, and cement use for fixation of the component. Cement procedures were described in six studies [6,7,14,15,29,30], and two studies described a mixed procedure of one cemented component and one non-cement component [28,31]. Numerous studies did not report or clearly describe the components or the surgical procedure [2,3,13,15,17-19,22,24,26,29,32]. Only a few of the studies controlled the number of surgeons performing the arthroplasties, thereby providing some control over the variation in the operating room procedures [17,23,24]. Therefore, the specific surgical details were widely varied among the study pool.

### Retrospective evidence

We identified five retrospective studies, and the summaries of these studies are found in Table 1. Three studies evaluated obesity effects on HHS score values over varied follow-up time points out to 20 years. A BMI value of >30 kg/m^2^ was related with a 4-9% lower HHS scores at follow-up than BMI values less than 30 kg/m^2 ^[14,24]. Despite HHS score differences, implant survival duration was similar by year 11. One study that assessed follow-up HHS scores did not find differences in improvement levels between non-obese and obese patients after the procedure [11]. This corresponded with no differences in revision rates or medical complications. Two other reports showed that patients with higher BMI values had 4-9% lower HHS scores at maximum follow-up time of 1–10 years [14,24], and significantly less range of motion during hip flexion, adduction, internal rotation and lower knee flexion values than patients with normal BMI values. Similar to the findings of McLaughlin and Lee [11], Yeung et al [24] reported no differences in the probability of 11 year in implant survival based on BMI.

**Table 1:** Retrospective studies of functional outcomes in obese and non-obese patients with total hip arthroplasty (THA)

Study	N Follow-up	Sample	Surgical Type & Components	Results
Braeken et al [2] (1997)	193 to 1 year; Retrospective Mean age 63.5 years	61% were women BMI was found for each patient	Surgical components not described; surgical type not described: data obtained from medical charts and mailings	While high BMI was related to high postoperative pain levels, BMI itself was not a strong contributor to the regression model for WOMAC functional score (parameter estimate value of 0.092).
Haverkamp et al [14] (2008)	411 Mean out to 20 years; Retrospective	69% were women; BMI groups were <25, ≥ 25 and >30 kg/m^2^ Mean ages were 64–66 years among groups	Anterolateral approach; Weber rotation THA System (Allopro) with cement;	HHS scores were progressively lower for higher BMI brackets at maximum follow-up time (91.6, 86.8 and 83.7 points; p = 0.02); revision rates were similar among BMI complication rates and groups
LeDuff et al [7] (2007)	770 2–10 years; Retrospective	17% were women BMI groups were < or ≥ 30 kg/m^2^ Mean age 49 years	Posterior approach; 30-37% of metaphyseal stem femoral components were cemented; Conserve Plus hip resurfacing prostheses were used	By 6.2 years of follow-up, UCLA scores for function and activity werelower in the obese patients than non-obese patients by ~8%; SF-12 physical component scores were also lower in the obese group (49.3 vs 51.4 points; p = 0.013); 5 year survivorship was 90.6% and 98.6% in patients with BMI <25 and 30 kg/m^2^, respectively.
McLaughlin & Lee [33]	285 10–18 years; Retrospective	51% were women; BMI groups were <30 or ≥30 kg/m^2^ Mean ages 54–57 years	Uncemented T-tap acetabular components (Biomet Inc.) and Taperloc femoral points in obese patients and from 53 to components were used by one surgeon; all were posterolateral approaches	By follow-up HHS ↑ from 52 to 89 89 points in non-obese patients, with no difference between groups; no differences in revision rates or complications occurred between groups
Yeung et al [24] (2010)	2,026 6.3 year mean follow-up; Retrospective	53% were female; BMI groups were <30 or ≥30 kg/m^2^	Cementless procedures used; components and approached were not described	HHS scores were lower for the obese compared to the non-obese patients at follow-up (89.9 vs 93.2 points; p < 0.001); HHS scores for function, activities, hip range of motion were lower in the obese group (all p < 0.05); survival rates were for the implants were similar at year 11 (95-96%)

THA = total hip arthroplasty; BMI = body mass index; EUROHIP = European Collaborative Database of Cost and Practice Patterns of Total Hip Replacement.

OR = odds ratio; RR = relative risk; HHS = Harris Hip Score; UCLA = University of California and Los Angeles (UCLA) activity scale; Medical Outcomes SF-12 = Short Form 12; WOMAC = Western Ontario and McMaster Osteoarthritis Index; VAS = visual analogue scale.

NS = not specified.

One study examined whether the functional and quality of life outcomes of hip resurfacing varied among non-obese and obese patients using data obtained from an institutional registry [7]. Data were obtained from clinical visits at four months, one year and annually after surgery, with follow-up times ranging from 2–10 years. In obese patients, post-operative HHS score were 3.2 points lower, UCLA sub-scores for “function” and “activity” were 4-7% lower than those of the non-obese patients. UCLA sub-scores of “walking” were not different between groups. Interestingly, survival of the hip implant was higher in the obese patients compared to the non-obese patients by year 5 (98.6% vs 90.6%).

### Prospective evidence

Table 2 summarizes the prospective studies that we identified. Six studies used HHS as primary endpoints [6,8,15,17,19,28], one study used OHS [16], one used the UCLA activity score [31], and six studies employed the WOMAC survey scores [8,18,26,28,34,35] as a main outcome. Six studies used actual functional measures and patient reported functional abilities [3,12,20,23,34,35].

**Table 2:** Prospective studies of functional outcomes in obese and non-obese patients with total hip arthroplasty (THA)

Study	N Follow-up	Sample	Surgical Type & Components	Results
Aderinto et al [15] (2005)	140 3 years; Prospective Follow-up	61% were women; groups were <30 or ≥30 kg/m^2^	Cemented prostheses; approaches or components were not described	At year 3, HHS scores ↑ from 44 to 90 points (non-obese) and from 42.5 to 85 points (obese), with no difference between groups; lower scores for stairs, sitting and putting on shoes-socks and range of motion were lower in the obese patients (p < 0.05).
Andrew et al [16] (2008)	1,421 5 years; Multi- center Prospective	62% were women; BMI groups were <30, 30- < 40 and ≥40 kg/m^2^ Mean ages were 69.1, 65.5 and 60.6 years	Anterolateral or posterior approaches were used; cemented Stryker Exeter femoral components and several different acetabular components	By year 5, OHS scores were best in the non-obese group and worst in the obese group (19.6 vs 25.6 points; p = 0.005), but no differences in the 5 year change in OHS scores existed. No differences in rates of revision, dislocations or medical complications existed.
Busato et al [3] (2008)	18,968 15 years; Multi- center prospective	sexes NS; BMI groups were <25, 25- <30 and ≥ 30 kg/m^2^	Surgical components not described; surgical type not described; Data were obtained from the Total Hip Registry (Switzerland)	High preoperative BMI was related with a dose-effect response with shorter unsupported walking time less normal stair climb, and shoe tying during the 15 year follow-up, despitesimilar pain relief across BMI brackets.
Chan and Villar [17] (1996)	166 to 3 years; Prospective cohort	59% were women BMI groups were <25, 25–29.9, 30–39.9, >40 kg/m^2^ Mean ages were 71.4, 69.0 and 68.1 years	Surgical components not described; surgical type not described	HHS scores were superimposed onto the Rosser Index Matrix (which ranks disability status); there were no differences in Rosser scores for disability among the BMI groups by year 3.
Chee et al [6] (2010)	108 5 years; Prospective cohort	41% were women; BMI groups were <35 kg/m^2^ or >35 (1 comorbidity) and >40 kg/m^2^	Anterolateral approach was used on all patients; 25.5% Charnley prosthesis (dePuy, Int.), 74.5% Lubinus SPII prosthesis (Waldmar-Link GmbH); cemented	Five year HHS were higher in non-obese than morbidly obese patients (91.8 vs 85.4 points; p < 0.0001) despite similar pre- operative scores; SF-36 subscores for physical functioning were lower in morbidly obese patients at year 5.
Dowsey et al [29] (2010)	471 1 year; Prospective follow-up	60% were women; BMI groups were <30, 30–39 and ≥ 40.0 kg/m2	Surgical procedures not described; surgical components not described; cement used varied across groups	Morbidly obese patients had a lower change in HHS function scores than obese and non-obese patients, respectively by year 1(11.5 vs 15.6 and 16.2 points, respectively); HHS were lowest in morbidly obese patients by year 1 (70.5 vs 79.8 and 80.8 points p = 0.03).
Gandhi et al [26] (2010)	707 hips I year Prospective cohort	59-66% were women; waist circumference was assessed for metabolic syndrome BMI ranged from 22.0 to 36.6 kg/m^2^ 64.8-66.2 years	Surgical components or procedures not described; patients were obtained from a registry	1 year WOMAC scores (pain, function) were highest in patients with 4 metabolic syndrome factors compared to those with fewer factors; regression B coefficients showed that obesity predicted 1 year WOMAC scores (B = 2.4 1.4-4.2; 95% CI).
Jackson et al [23] (2009)	2,026 mean of 5 years; Prospective cohort	77% were women; BMI groups were Mean ages were 68 and 63 years	Posterior surgical approach; ABG2 Stryker cementless femoral and acetabular components	HHS were lower in obese vs non-obese patients at follow-up (89.9 vs 93.2 points); HHS functional scores were also lower in the obese group (29.6 vs 31.0 points); hip flexion, adduction and internal rotation ranges were less in the obese vs non-obese patients. HHS pain scores were not different between groups.
Judge et al [18] (2010)	1,327 1 year EUROHIP Study of 20 orthopedic centers	56% were women; BMI groups were <30, 30–39 and ≥40 kg/m^2^ Ages <50 to ≥ 70 years	Surgical components not described; surgical type not described	Median WOMAC scores were highest in the morbidly obese group pre-THR, (68.1 vs 61.5 and 57.6 points). but the 1 year change in WOMAC score was highest in the morbidly obese group (median score change of 56.1 vs 33.3 and 37.5 points); morbidly obese patients showed a ↑ OR of “returning to normal” (functionality) than the other groups.
Lubbeke et al [28] (2007)	435 5-year; Prospective cohort	53-55% are women; BMI groups were < or ≥ 30 kg/m^2^ Mean ages were 68 72 years	85% of patients had mixed components (1 cemented,1 non)	Obesity was related with worse outcomes after revision than primary THA by year 5 (lower HHS scores: 76.7 vs 88.1 points; lower WOMAC function scores 61.6 vs 70.0 points). BMI was related to the mean difference of HHS scores for primary and revision THA (R coefficient = −1.0 [−0.1 to −1.95% CI]).
Lubbeke et al [8] (2007)	2,495 5-year; Prospective cohort	48.7-57.5% women; 8.5-9% were revisions; BMI groups were < or ≥ 30 kg/m^2^ Mean age 69 years	95% were lateral THA approach, 86% used Morscher press-fit uncemented actetabular component and Muller straight stem cobalt chromium femoral component	BMI of ≥30 kg/m^2^ was related with a RR of 3.7-4.0 for revisions, 9.1-12.5 for dislocations and1.9-8.0 for infections in obese compared to non-obese men and women. By year 5, HHS were 87.8 and 79.6 points in non- obese and obese women and 90.5 vs 87.4 points in non-obese and obese men; WOMAC function scores were 14.7% and 8.0% lower in obese women and men thantheir non-obese counterparts.
Lubbeke et al [30] (2008)	204 5-year; Prospective cohort	50-7.9% were women; BMI groups were < or ≥ 30 kg/m^2^ Age range <50 to ≥80 years	Cemented acetabular cups were used in 67% and 80% of obese and non-obese patients	HHS were 82.8 ± 14.7 and 71.4 ± 17.0 points in the non-obese and obese patients by year 5. Surgical revisions were performed at 92 and 125 months in obese and non-obese groups, respectively. The adjusted hazard ratio for occurrence of infection, dislocation or re-revision increased from 1.0 (BMI < 25) to 1.5 (BMI 25–29.9) to 4.5 (BMI 30–34.9) to 10.9 (BMI ≥35.0).
Lubbeke et al [31] (2010)	503 5 or 10 years; Prospective follow-up	58% were women; BMI groups were <25, 25–29.9 and ≥ 30 kg/m^2^	Hybrid prosthesis; Morscher press fit uncemented cup and cemented cobalt-chromium stem (Zimmer); alumnia ceramic head, and a ceramic- polyethylene surface	At year 5, HHS ↓ with each rogressively higher BMI group (91.4, 88.4 and 85.1 points; p = 0.019). At year 10, HHS tended to be lower in patients with BMI ≥30 kg/m^2^ compared with those with BMI <25 and 25–29.9 kg/m^2^ (83.6 vs 87.3 and 87.1 points; p = 0.08); more obese patients had low UCLA scores and more non-obese patients had higher UCLA scores.
Moran et al [19] (2005)	800 6–18 mo; Prospective follow-up	61% women; BMI groups were <25, 25–29.9, 30–39.9 ≥40 kg/m^2^ Mean age was 68 years	All were anterolateral approach surgeries; components were not described	For every 1 point increase in BMI, HHS scores dropped by 0.25 by month 6 and by 0.35 by month 18 post-surgery. No BMI effect on early failure of THA was found.
Naylor et al [22] (2008)	198 1 year; Prospective observational	56% were women; BMI groups were <30 or ≥30 kg/m^2^ Mean age 67 years	Surgical components not described; approaches not described	Obese patients had smaller increases in timed mobility than non-obese patients (0.23 m/s slower on 15 m walk time) and the timed up and go test (3.1 sec slower) at year 1; WOMAC scores for function and pain were worse in obese than non-obese patients by year 1.
Singh et al [12] (2009)	2,687 2–5 years; Prospective cohort of revision THA	53-54% were women; BMI brackets were <25, 25–29.9, 30–39.9 ≥40 kg/m^2^ Mean 5 year age was 65 years	Surgical components not described; approaches not described	At year 2, the OR for complete dependence onwalking/gait aids was 2.0 (vs 0.9 for BMI 25–29.9); moderate to severe activity limitation was predicted by high BMI. The OR of reporting difficulty in 3 of 7 mobility and functional tasks ↑ from 1.2 to 2.7 with increased BMI from 25–29.9 to 40 kg/m^2^, by year five, the OR increased to 1.3 and 3.0 in these same BMI brackets (all p < 0.01).
Søballe et al (1987) [20]	125 5 years; Prospective follow-up	A weight index was calculated as < or > 120% of pre-surgical weight; analyses were also performed using weight brackets of < or > 80 kg; Mean age at follow-up 70 (28–89) years	All were posterolateral approach surgeries; Lubinus prostheses were used and fixed with gentamicin impregnated radiopaque PMMA; One surgeon performed all hip replacements	Walking ability, defined using the Charnley scoring system was lower in patients with a weight index >120 pre-surgery, but similar to patients with indexes <120 by year 5 (4.9 vs 5.0 points; p = NSig).
Stickles et al [13] (2001)	5921 year; Prospective follow-up	56% were women; BMI brackets were <25, 25–29.9, 30–40 >40 kg/m^2^ Mean age 69 years	Surgical components or procedures not described; patients were obtained from a registry	By year 1, stair ascension and descension difficulty was reported in 86-88% of very obese patients compared with 46-55% of non-obese patients; BMI did not correlate with change in WOMAC scores (31.8 and 35.9 points, in non-obese and very obese patients, respectively; p > 0.05).

THA = total hip arthroplasty; BMI = body mass index; EUROHIP = European Collaborative Database of Cost and Practice Patterns of Total Hip Replacement.

OR = odds ratio; RR = relative risk; HHS = Harris Hip Score; UCLA = University of California and Los Angeles (UCLA) activity scale; Medical Outcomes SF-12 = Short Form 12; WOMAC = Western Ontario and McMaster Osteoarthritis Index; VAS = visual analogue scale.

NSig = non-significant.

### Harris hip score

HHS scores and functional sub-scores were captured at a variety of time points ranging from one to five years. In one study, sub-scores of HHS were reported in non-obese and obese patients with THA by three years post-surgery [15]. Sub-scores of walking distance, climbing stairs, putting on shoes and socks, sitting and hip range of motion were 4-25% lower in the obese group at follow-up. Despite mean improvements in HHS and mobility, the cohort tended to gain weight by year three. Another study tracked functional and clinical outcomes in a group of morbidly obese patients with THA out to year five; HHS scores improved significantly in all patients by month six [6]. While the HHS scores were higher in non-obese patients by year five, the absolute improvement in scores were not different based on obesity status (52.0 vs 48.1 point change). Also, the morbidly obese patients had more peri-operative complications (12 vs 3 complications) and the five year survival was 90.9% and 100% in the obese and non-obese patients.

Both sex and revision status may be important factors influencing HHS outcomes. For example, HHS scores were evaluated among patients with a primary or revision THA in a Swiss cohort [28]. Patients with revisions had a relative risk of 1.67 for being obese, and BMI was found to be a stronger inverse predictor of HHS after revision procedures than primary procedures by year five. In a related study by the same author [8], HHS scores were collected in Swiss patients five years after THA procedures and found that 81% and 70% of non-obese and obese patients had good to excellent outcomes, defined as a HHS score ≥80 points. These poorer outcomes were magnified in women than men. For example, HHS scores in non-obese and obese women were 87.8 and 79.6 points respectively, and were 90.5 and 87.4 points in non-obese and obese men.

Among predictive and correlational studies, Moran et al [19] examined whether BMI predicted HHS scores at follow-up times of six and 18 months post-THA. While no BMI-specific HHS data were presented, the authors reported a small but significant multiple regression coefficient for BMI on the model for the follow-ups HHS. This translated to a reduction in HHS by 0.25-0.35 points per one point increase in BMI value. Another correlational study that we included did not report actual HHS scores, but superimposed HHS scores onto the Rosser Disability Index matrix to generate a “quality of life” score as a method of estimating functional disability [17]. The findings revealed that the median quality of life scores were not different among patients with low and high BMI values at one and three years of follow-up [17]. The authors concluded that progressively higher BMI values up to 40 kg/m^2^ were not related with these disability estimates.

### WOMAC scores

One study examined the predictive value of one to four factors relating to the metabolic syndrome on WOMAC scores before and at one year after THA [26]. These factors included large waist circumference (>102 cm men, >88 cm women), elevated triglycerides and low high density lipoprotein cholesterol, high blood pressure and elevated fasting blood glucose. BMI was calculated for each patient. Regression analyses were performed to determine the effect of the number of metabolic syndrome factors on WOMAC scores by year one. The regression beta coefficient of having 4 metabolic factors on prediction of WOMAC scores was 16.1 in this obese patient group compared with the beta coefficient of 0.6 in non-obese patients with one factor. The coefficient for obesity alone was 2.4 (p = 0.03).

A study that analyzed data from twelve European countries showed that one year WOMAC scores are related to BMI [18]. Median baseline WOMAC scores were higher in morbidly obese and obese patients compared with non-obese patients (68.1 and 61.5 vs 57.6 points) but the median change in WOMAC scores was highest in the morbidly obese group by year one (56.1 vs 33.3 and 37.5 points; p = 0.012). In this cohort, the multivariate odds ratio of returning to normal functional status was 3.1 compared to non-obese patients.

Lubbeke et al. performed two hospital-based cohorts examining the 1) five year outcomes in obese men and women with THA [8], and 2) the effects of comorbidities and age after primary and revision THA [28]. In the first study, sex differences in survey outcomes were analyzed in a cohort of patients with THA. The crude incidence rate was 4.7 times higher for infection, and the incidence rate was 2.3 times higher for dislocation in obese patients compared to non-obese patients. Five-year WOMAC function sub-scores were 11% lower in obese women than men and 8% lower in non-obese women than men. Within each sex group, obese women and men had 8-11% lower WOMAC function sub-scores than their non-obese counterparts. In the second study, patients with primary and revision procedures were administered the WOMAC at five years [28]. of note, the patients with revisions were more often obese than not. The WOMAC function sub-score was reported to be lower in obese patients with revision THA compared with non-obese patients (61.6 vs 70.0 points). This difference was deemed small but clinically significant.

A sample of Australian patients with primary unilateral THA was analyzed for differences in WOMAC responses by year one [34]. The obese patients reported improvements in WOMAC function subscores from 44.6 to 23.3 points while the non-obese patients demonstrated improvements in this same subscore from 40.7 to 13.7 points. Hence, the non-obese patients showed a greater relative functional improvement than obese patients at year one. Stickles et al [35] prospectively assessed WOMAC responses in persons with THA across the BMI spectrum: <25, 25–20, 30–35, 35–40 and >40 kg/m^2^. At one year after surgery, WOMAC scores were progressively less with each higher BMI bracket. There were no differences among BMI brackets in the total complication rate, medical complication rate or orthopedic complication rate.

### Oxford hip scores (OHS)

One study presented responses to the Oxford Hip survey in groups with low and high BMIs. Andrew et al. [16]. compared the OHS in non-obese, obese and morbidly obese patients pre-surgery, three months and annually until year five. By year three, the average OHS was highest in the non-obese group and lowest in the morbidly obese group (19.6 ± 8.6 vs 23.5 ± 11.4 points); this pattern was maintained out to year five. However, the five year change in absolute OHS was not different based on BMI, indicating comparable responsiveness to the procedure over the long term. There were no differences in the rates of perisurgical medical complications or long term femoral stem position, femoral osteolysis or implant survival.

### UCLA activity score

A prospective study that compared the osteolysis rates and patient satisfaction after THA was conducted by Lubbeke et al [31]. The patient activity level was assessed using the UCLA survey at the pre-surgery time point and at years five and ten after THA. Patients were stratified into non-obese, overweight, and obese groups based on BMI. While the mean UCLA scores were similar between groups at follow-up, the 6.8% of obese patients achieved a high activity UCLA score compared to 11.7% overweight and 14.4% of normal weight groups. This finding was corroborated with lower follow-up HHS in the obese group compared to the remaining groups. Obese patients had poorer pain scores and an odds ratio of 1.44 of developing femoral osteolysis whereas the normal weight had an odds ratio of 2.64 compared to the reference overweight group.

### Functional tests

Several studies assessed functional capacity with walking tests, stair climbing, and timed functional tests. In an early study, walking ability was a primary functional five year endpoint for a Danish cohort of patients with THA [20]. Radiographic and surgical outcomes were measured in addition to walking ability. The Charnley criteria [36] were applied to walking ability (6 points = walking, no limp; 5 points = extensive walking, with or without cane, with limp; 4 points = moderate walking with one crutch or cane; 3 points = restricted walking with crutches; 2 points = wheelchair transfer activity; 1 point = bedridden) before and after THA. Patients were stratified based on a weight index (< or >120% of normal weight for height and sex) or by body weight (<80 kg or >80 kg). Pre-operatively, walking ability scores were different based on % of normal weight (1.8 vs 2.3 points in heavy versus non-heavy patients, respectively; p < 0.05) but not absolute body weight (2.2 points for both groups). Five years post-THA, Charnely walking scores were not different based on either expression of body size. While surgical blood loss was greater in heavy patients compared with non-heavy patients, there were no significant differences in operation time, medical complications or loosening of the implant based on weight.

Naylor et al [34] in Australia, performed short functional tests in obese and non-obese patients before and at various time points out to year one. These tests included a 15-meter walking test and a timed-up-and-go (TUG) test where an individual rises from a chair and returns to the chair. While obesity did not preclude improvement in 15 meter walking speed, the trajectory of change was lower in the obese group by year one, and final walking speeds were 1.37 m/s and 1.14 m/s in non-obese and obese patients, respectively. Similarly, improvements in TUG test time were slower in the obese group by year one (final TUG times were 9.2 sec and 12.4 sec in the non-obese and obese patients). The approximate 30% an 42% improvements in walk and TUG times were not different based on obesity status. There was a 2.8% greater chance that obese patients would use a walking aid at follow-up compared to non-obese counterparts.

Difficulty ascending and descending stairs was one endpoint in a prospective study of American patients hip or knee arthroplasty [35]. Among the patients with THA, and it was shown that the percentage of patients in each progressively higher BMI bracket (<25 to >40 kg/m [2]) reported difficulty with descending and ascending stairs. This functional pattern was corroborated with self-report WOMAC data, which showed lower scores in the one year total WOMAC score and related physical component subscore in severely obese patients compared to non-obese patients. Total medical complication rate was not different across the BMI spectrum. However, a major limitation to the study was that the specific technique used to capture this stair climbing information was not presented in the methods.

In a cohort of American patients with revision THA, activity limitation and dependence on walking aids was assessed by Singh et al [12] at years two and five post-THA. The evidence revealed that the odds ratio of developing moderate-severe physical activity limitation increased from 1.2 to 2.7 in overweight and morbidly obese patients. Severe obesity increased the two and five year odds ratios of developing complete dependence on walking aids to 2.0 and 2.7 compared with the reference of normal weight.

Busato et al [3] collected a series of functional measurements in a cohort of Swiss patients with THA at periodic follow-ups, including walking distance without support, hip flexion range, stair climbing and putting on shoes and socks. Data were reported as the percent of patients who were able to achieve walking >60 minutes, hip flexion range >90 and capacity for normal stair climb and donning of footwear. Irrespective of time point, fewer obese patients were able to walk >60 minutes without support and this percentage continued to lowered 27.2% from year three to year 12 compared with the 18.5% fewer non-obese patients. This same pattern occurred with stair climbing and with tying shoes by year 12. Interestingly, the obese patients increased the hip flexion range of motion by 7.8%, whereas the non-obese group lost 11.6% of hip flexion motion by year 12. In all three BMI strata, pain relief was achieved in the majority of patients and was largely maintained by year 12.

Hip range of motion was assessed in non-obese and obese patients with cementless THA at a median follow-up time of 5.1 years in addition to pain ratings, survey scores and implant survival. Despite equivalent implant survival rates between groups, obese patients achieved 9.4 less range of movement about the hip than non-obese patients with flexion, 2 less adduction and 2.8 less of internal rotation. Interestingly, pain relief and patient satisfaction with the procedure were similar between groups at follow-up.

### Overview of the findings

Of the evidence included in this review, the surgical component, cementation or surgical approach did not appear to have any consistent effect on long term functional outcomes in the obese patient. Of the 23 studies, 16 reported that obesity was associated with lower hip function survey scores at follow-up, whereas four studies did not. Five studies revealed negative long term effects of obesity on various tasks of physical function, and one study noted no adverse influence of obesity on walking ability. While obesity does not necessarily preclude functional improvements after surgery, the functional gains achieved and the self-reported functional ability generally are lower than those attained by non-obese patients. Thus, evidence indicates that there is significant pain relief and patient satisfaction after THA in obese patients, whereas these patients fall short of achieving the same long term functional level as their non-obese counterparts. Hence, in a time frame of as short as one year after THA, obese patients are already at a functional deficit compared to non-obese counterparts.

#### Potential explanations for functional outcomes in the obese patient

Some postulated mechanisms underlying this finding include fear of movement and fear avoidance of physical activities, poor skeletal muscle quality, elevated perceived effort with physical tasks, and maintenance of pre-surgical physical activity patterns. Our research laboratory has recently shown that obese patients fear pain induced by physical activity and movement, and this corresponds to lower self-reported physical abilities with tasks related to mobility (e.g., walking, stair climbing, body transfers and recreational activities that involve running, start and stop motions and jumping) [37,38]. Despite a paucity of literature addressing the interactions of pain, obesity and joint function over the long term, obese patients can achieve high satisfaction and pain relief and still not increase participation in physical activities at home and in the community. Whether this is due to residual fear of movement and avoidance of activities that may trigger pain is unknown.

Muscle strength rather than muscle mass may be a more clinically relevant determinant of functional status in the older population [39]. Adipose tissue itself contributes to muscle loss through chronic, low grade production of inflammatory cytokines, adipokines and free fatty acids [40]. These chemicals slowly erode muscle mass in the older obese adult, unfavorably shifting the ratio of muscle mass to fat mass. In addition, existing muscles become infiltrated by fat [41], thereby lowering muscle quality and functional ability of the muscle tissue. Residual post-operative strength deficits and fat infiltration may occur in the hip flexors (15% strength difference compared to non-repaired hip) out to two years [42]; these deficits may contribute to lower functional ability with tasks that activate these muscles. Hence, independent from the improvements in pain and quality of life after a total hip replacement, the obese patient may not achieve the same level of function as non-obese patient in part due to skeletal muscle quality, especially if the patient does not change physical activity patterns to activate loading bearing muscle groups after THA.

The level of perceived difficulty and effort with physical activity is a critical issue for obese individuals, especially those with BMI ≥ 40 kg/m^2^. Morbidly obese patients have poor cardiac, metabolic and ventilatory efficiency and have lower compensatory hyperventilation with exercise [43]. A given exercise workload may require a lot of energy to move heavier limb segments against gravity in the morbidly obese individuals (manifested as lower mechanical efficiency) [44,45]. Self-reported fatigue is higher with increasing BMI [46]. Mobility related tasks, such as short and long distance timed walking tests, stair climb. TUG tests are consistently more challenging and take longer to perform with increasing BMI [47]. Musculoskeletal pain may occur at high intensity activities and may be the limiting factor and not true muscle fatigue [45]. Collectively, the higher perception of muscular effort and discomfort of dyspnea may discourage this population from regularly engaging in physical activities.

While quantitative evidence of physical activity patterns after THA in obese adults is sparse, Donovan et al [48]. reported that patient perceptions of physical activity improved by one year after surgery. A total of 43% and 9% of the patients felt that their activity level improved “a lot more” and “a little more” since prior to joint replacement, respectively. The majority of the referenced studies, however, showed that weight gain or maintenance typically occurs after THA, inferring that physical activity levels were not dramatically altered from pre-surgical levels. It has been postulated that with the resolution of hip pain and decreased need for analgesics, normal appetites and caloric intake returned and counteracted any potential effect of increased physical activity in the obese patient [49].

#### Does weight loss after THA improve long term functional outcomes?

The assumption that THA would reduce hip pain, and therefore foster increased physical activity and weight loss in the obese patient is not presently substantiated by the limited evidence. In fact, several studies report maintenance of current weight [48] or continued weight gain (ranging from 2.5%-3.6% increase in BMI) one to two years after THA [49-51]. Data suggest that ≥75% of patients experience weight gain over three years [15]. This trend occurs in men and women, irrespective of pre-surgical BMI [50]. However, in one prospective study, the average weight gain by one year was 2.8 kg, with the change occurring primarily in women [52]. In another study, non-obese and obese patients did not lose weight, while overweight patients gained a significant amount of weight after surgery [53]. Post-surgical BMI values at follow-up were independent of improvement in mobility using WOMAC score [51] or self-reported improvements in mobility [54]. We surmise that the majority of patients undergoing primary THA will increase their BMI given sufficient follow-up time, irrespective of the outcome [51]. Further investigation of strategies that can be used to encourage perioperative weight loss and slow, safe weight loss after surgery will be important for functional change and preservation of the joint prosthesis lifespan.

#### Clinical implications

Obese patients with THA are less functional than non-obese counterparts. If over time the obese patient fails to modify lifestyle, sarcopenic obesity worsens as a spiral of deconditioning and functional dependence develops. The ability to generate muscle strength and power will decline without participation in physical activity. As the obese patient ages and the negative lifestyle behaviors remain, obesity-related comorbidities will worsen. Additional joint pains will develop and the joint implant will ultimately fail earlier in life. Uncontrolled obesity after THA will result in costly and complicated surgical revision procedures. This is a serious issue, because the number of THA performed earlier in obese patients is growing rapidly in the U.S. Possible solutions to this combat this problem include: 1) a strong emphasis on aggressive rehabilitation after THA that focuses on long term maintenance of skeletal muscle quality and mass, and 2) consistent, safe monitored weight loss over the months following the surgery to facilitate physical function and longevity of the joint implant.

#### Limitations to the evidence

There are several limitations that deserve comment. First, the determination of “physical function” varied widely across the studies. In this population, there is not yet a standard test battery performed before and after surgery. Second, the reviewed studies typically did not report severity or frequency of other musculoskeletal pain that might be affected by THA. This issue is important, as the hip joint lies centrally to the lower body kinematic chain. Correction of hip pain may improve gait, posture and ambulation, thereby reducing other joint symptoms in the spine and knee. We assume that correction of pain and disability in the hip is the mechanism underlying functional improvement, particularly in obese patients, but we are neglecting to address whether THA has wider reaching effects on overall joint mechanics and pain symptoms.

A substantial attrition rate occurred in many studies. Attrition creates selection bias of the population, and in many studies it was not clear how missing data were handled. In some cases, only completers were included in the analysis. Interpretation of long term results is confounded by the likelihood of selection bias. The lack of emphasis on clinical importance has led to misconceptions and disagreements about the interpretation of the results of clinical trials and a tendency to equate statistical significance with clinical importance. In some instances, statistically significant results may not be clinically important and, conversely, statistically insignificant results do not completely rule out the possibility of clinically important effects [55].

#### Future directions

The National Health and Nutrition Examination Survey III data show that lower body functional impairment is becoming more prevalent in obese individuals; among obese persons, 36.8% reported disability in 1994, whereas 42.2% reported disability and functional impairment in 2004 [56]. Development of a battery of functional tests of hip flexibility, muscle testing and mobility should be standard practice for THA research to help predict functional success of the surgery. A comprehensive pain and fear assessment will help determine how systemic pain relates to the symptoms and changes in the hip joint. Prospective biomechanical assessments of lower body kinematics in the surgical and non-surgical limb would be very useful in tracking the long term joint motion and physical capabilities. Psychiatric [57], psychosocial [58] and behavioral traits [59] or genetic phenotypes [60] associated with severe obesity should be factors to track in future outcomes studies. There is a dire need for identification of specific exercise, psychosocial, pain management and nutritional interventions that effectively enhance gains made in physical function over the long term. Finally, it would be clinically important to determine what amount of weight loss maximizes implant lifespan.

## Conclusions

THA confers significant pain reduction and improvement in quality of life irrespective of BMI. While functional improvement occurs after THA, available evidence indicates that obese patients are less likely to attain the same level of physical function over the long term. Uncontrolled obesity is related to worsening of comorbidities and excessive health care costs over the long term. Aggressive and sustainable rehabilitation strategies that include physical exercise, psychosocial components and behavior modification may be highly useful in maximizing and maintaining weight loss after THA. Furthermore, this type of rehabilitation may also reduce the overall health care burden.

## Competing interests

The authors declare that they have no competing interests.

## Authors’ contributions

HV primary writer. MBH text contributor, editing. PFG text contributor, editing. RV text contributor, editing. ANS collection of scientific papers, text contributor. BPC text contributor, editing. KRV text contributor, editing. All authors read and approved the final manuscript.
